# Herbal Medicine, Gut Microbiota, and COVID-19

**DOI:** 10.3389/fphar.2021.646560

**Published:** 2021-07-07

**Authors:** Ziqi Chen, Yiwen Lv, Huachong Xu, Li Deng

**Affiliations:** ^1^ College of Traditional Chinese Medicine, Jinan University, Guangzhou, China; ^2^ Medical College, Sun Yat-sen University, Guangzhou, China

**Keywords:** herbal medicine, gut microbiota, COVID-19, SARS-CoV-2, intestinal mucosal barrier

## Abstract

Coronavirus Disease 19 (COVID-19) is a respiratory disease caused by severe acute respiratory syndrome coronavirus 2 (SARS-CoV-2), which has grown to a worldwide pandemic with substantial mortality. The symptoms of COVID-19 range from mild flu-like symptoms, including cough and fever, to life threatening complications. There are still quite a number of patients with COVID-19 showed enteric symptoms including nausea, vomiting, and diarrhea. The gastrointestinal tract may be one of the target organs of SARS-CoV-2. Angiotensin converting enzyme 2 (ACE2) is the main receptor of SARS-CoV-2 virus, which is significantly expressed in intestinal cells. ACE2 links amino acid malnutrition to microbial ecology and intestinal inflammation. Intestinal flora imbalance and endotoxemia may accelerate the progression of COVID-19. Many herbs have demonstrated properties relevant to the treatment of COVID-19, by supporting organs and systems of the body affected by the virus. Herbs can restore the structure of the intestinal flora, which may further modulate the immune function after SARS-CoV-2 infection. Regulation of intestinal flora by herbal medicine may be helpful for the treatment and recovery of the disease. Understanding the role of herbs that regulate intestinal flora in fighting respiratory virus infections and maintaining intestinal flora balance can provide new ideas for preventing and treating COVID-19.

## Introduction

Coronaviruses are mainly divided into four genera, alpha (α), beta (β), gamma (γ), and delta (δ), which can infect humans and a variety of animals. The seven human coronaviruses (HCoV) that have been discovered are HCoV-229E, HCoV-NL63, HCoV-OC43, HCoV-HKU1, Severe acute respiratory syndrome coronavirus (SARS-CoV), Middle East respiratory syndrome coronavirus (MERS-CoV), and SARS-CoV-2. Coronaviruses can cause respiratory as well as gastrointestinal infections in humans and animals (Su et al., 2016). In addition to the respiratory system, the digestive tract is most commonly affected by coronavirus infections. The symptoms mainly include abdominal pain, nausea, vomiting, and diarrhea. Human gastrointestinal cells were highly susceptible to MERS-CoV, and the virus was able to maintain their replication robustly in small intestine cells (Xiong et al., 2020). About 1/3 of patients with Middle East respiratory syndrome (MERS) have gastrointestinal symptoms (Matoba et al., 2015; Corman et al., 2018). SARS-CoV-2 is an enveloped, positively charged single-stranded RNA virus belonging to the genus Coronavirus. SARS-CoV-2 is highly homologous to SARS coronavirus, and its nucleic acid sequence similarity reaches 70%. Similar to SARS virus infection, respiratory tract infection symptoms such as fever and cough are one of the most common clinical manifestations of COVID-19 patients (Zhou et al., 2020). Still, a considerable number of patients will also have gastrointestinal symptoms such as diarrhea (Zhang et al., 2020a).

Angiotensin-converting enzyme 2 (ACE2) is the primary receptor of the SARS-Cov-2 virus. ACE2 is significantly expressed in alveolar type II cells and intestinal cells (Hashimoto et al., 2012). Current studies believe that the gastrointestinal tract is one of the target organs of SARS-CoV-2 (Jin et al., 2020a). The positive viral RNA test in the stool of COVID-19 patients also confirms this to a certain extent and suggests SARS-CoV-2 transmission through the fecal-oral route. It undoubtedly poses a further challenge to the prevention and control of the COVID-19 epidemic. Although the respiratory symptoms related to COVID-19 have attracted significant attention, gastrointestinal symptoms are often overlooked, which can easily lead to a missed diagnosis. This review summarizes COVID-19, SARS, and MERS's clinical features associated with gastrointestinal symptoms, the related mechanisms of changes in the intestinal flora, and related herbs that can regulate intestinal flora. Understanding the gastrointestinal symptoms and possible mechanisms of COVID-19 is of great clinical significance for the early diagnosis, treatment, and control of the disease.

## Clinical Features of Coronavirus Disease 19, Severe Acute Respiratory Syndrome, and Middle East Respiratory Syndrome With Gastrointestinal Symptoms

**FIGURE 1:**
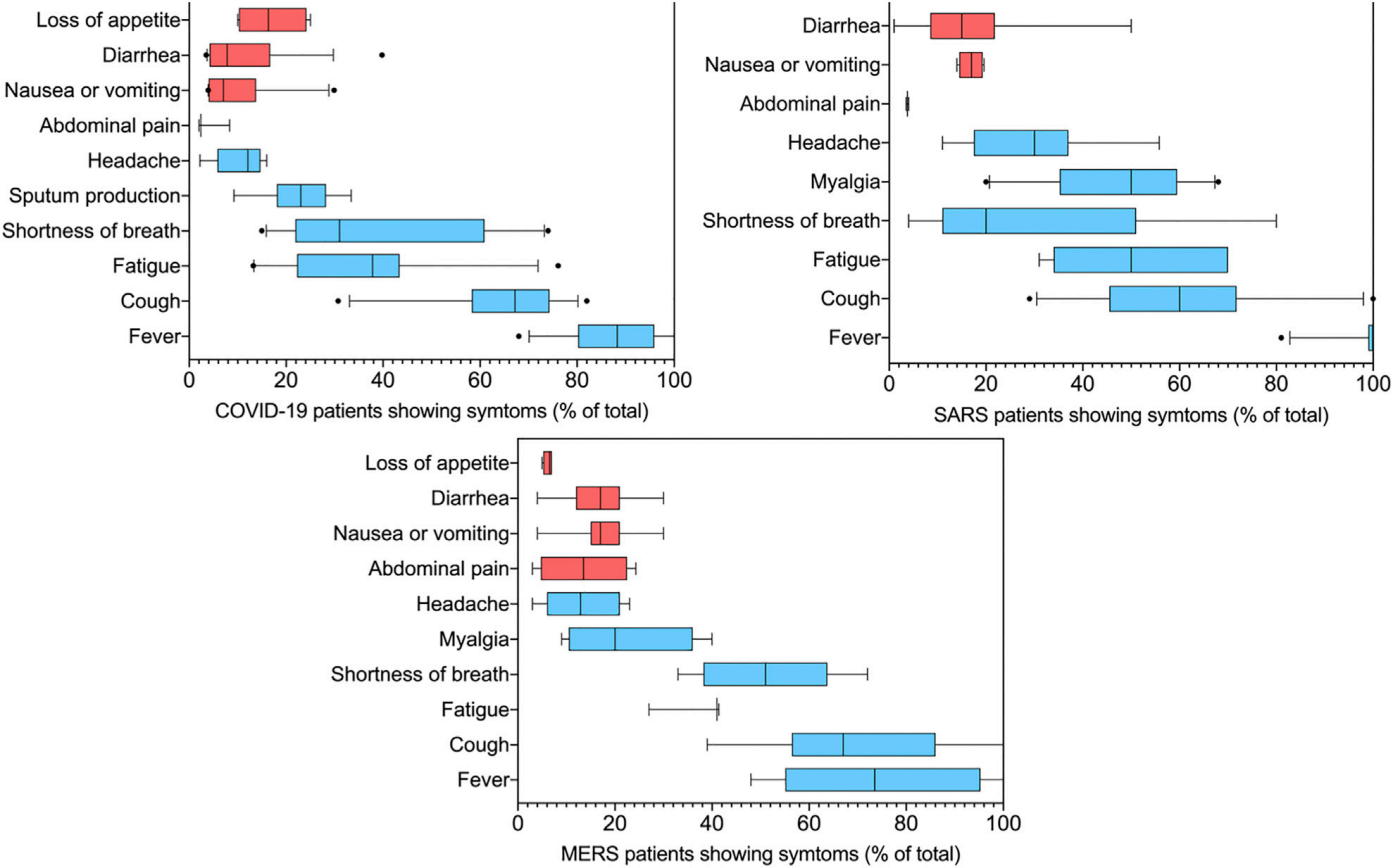
Summary of symptoms feature of COVID-19, SARS, and MERS. The data is the summary of 16 independent reports involving a total of 7322 COVID-19 patients, 10 independent reports involving a total of 1299 SARS patients, nine independent reports involving a total of 783 MERS patients. The red bars are those associated with gastrointestinal problems. In the box plots, the boundary of the box closest to zero indicates the 25th percentile, a black line within the box marks the median, and the boundary of the box farthest from zero indicates the 75th percentile. Whiskers above and below the box indicate the 10th and 90th percentiles. Points above and below the whiskers indicate outliers outside the 10th and 90th percentiles.

COVID-19 is mainly manifested by fever, fatigue, and dry cough, but some patients have abdominal pain, nausea, vomiting, diarrhea, and other symptoms. According to relevant reports on COVID-19 symptoms, the number, range, and severity of COVID-19-related symptoms may vary from person to person. Overall, our symptom analysis of independently published studies (Booth et al., 2003; Chan et al., 2003; Choi et al., 2003; Lee et al., 2003; Peiris et al., 2003a; Peiris et al., 2003b; Poutanen et al., 2003; Rainer et al., 2003; Tsang et al., 2003; Zaki et al., 2012; Assiri et al., 2013a; Assiri et al., 2013b; Memish et al., 2013; Al-Tawfiq et al., 2014; Saad et al., 2014; Noorwali et al., 2015; Oboho et al., 2015; Yin and Wunderink, 2018; Cattelan et al., 2020; Chen et al., 2020; Cummings et al., 2020; Guan et al., 2020a; Guan et al., 2020b; Huang C. et al., 2020; Li et al., 2020a; Qian et al., 2020; Shi S. et al., 2020; Suleyman et al., 2020; Wang D. et al., 2020; Wang Z. et al., 2020; Xu X. et al., 2020; Zhang et al., 2020a; Zhou et al., 2020) involving thousands of people shows that a small proportion but large number of patients experience gastrointestinal problems (Figure 1).

Among the 1,299 hospitalized patients with SARS included in the statistics, 514 (39%) developed gastrointestinal symptoms, included diarrhea (7–20%) (Booth et al., 2003; Chan et al., 2003; Peiris et al., 2003b; Tsang et al., 2003), there are also reports that a 50% (Poutanen et al., 2003) probability of appearing, nausea or vomiting (12–20%) (Lee et al., 2003; Yin and Wunderink, 2018). Similarly, in every two of the MERS inpatients, a gastrointestinal symptom occurs. Including loss of appetite (4–6%) (Assiri et al., 2013b; Oboho et al., 2015), nausea or vomiting (7–28%) (Assiri et al., 2013a; Oboho et al., 2015; Yin and Wunderink, 2018), abdominal pain (3–24%) (Assiri et al., 2013b; Memish et al., 2013) and diarrhea (5–30%) (Al-Tawfiq et al., 2014; Saad et al., 2014), the incidence of diarrhea was also reported as 75% (Memish et al., 2013). Consistent with SARS-CoV-1 and MERS-CoV, among a total of 7,322 COVID-19 patients, 1,104 patients suffered gastrointestinal problems, as high as 15% of cases. According to the report, 9–26% (Qian et al., 2020; Suleyman et al., 2020) patients loss their appetite (median 17%), 2–31% (Chen et al., 2020; Huang C. et al., 2020) had diarrhea (median 9.8%) with an outlier of the 40% (Zhang et al., 2020a) and 4–30% (Li et al., 2020b; Xu X. et al., 2020; Zhou et al., 2020)suffered from nausea or vomiting (median 6%). Incidence of gastrointestinal complaints, vomiting, and diarrhea caused by SARS-CoV-2 is similar to SARS-CoV-1 and MERS-CoV. Patients in severe or critical condition have the highest incidence of diarrhea (Guan et al., 2020b). In rare cases (Lee et al., 2020; Pan et al., 2020), diarrhea is the only symptom of COVID-19. The autopsy study of patients who died of COVID-19 also found that the small intestine showed segmental dilation and narrow changes (Wichmann et al., 2020). It is shown that the SARS-CoV-2 attacks not only the lungs but also the GI track. Theoretically, SARS-CoV-2 can bind to the angiotensin-converting enzyme 2 (ACE2) receptors in the intestinal cells, thus causing gastrointestinal disease and gastrointestinal symptoms such as abdominal pain and diarrhea.

## Effect of Severe Acute Respiratory Syndrome Coronavirus 2 on Human Intestinal Tract

### Severe Acute Respiratory Syndrome Coronavirus 2 Infection Association With Intestinal Flora Dysbiosis and Intestinal Barrier Disruption

At present, SARS-CoV-2 has been found and isolated in stool samples of patients with COVID-19 (Tang et al., 2020; Mohan et al., 2021), and changes in intestinal flora have been found in COVID-19 patients with gastrointestinal symptoms (Dhar and Mohanty, 2020). It is suggested that SARS-CoV-2 may cause intestinal flora imbalance while causing lung infection. The intestinal flora is composed of bacteria, viruses, fungi, and archaea. Viral infections can cause changes in the composition of intestinal flora, thereby causing intestinal barrier disfunction (Eckburg et al., 2005; Qin et al., 2015; Thaiss et al., 2016; Deng et al., 2020). Intestinal flora has a closely relationship with the expression of tight junction proteins in the intestinal epithelial cells. The dysbiosis of intestinal flora is accompanied by decreased expression of intestinal tight junction proteins such as Claudin-1, Occludin and ZO-1, leading to the disruption of intestinal barrier. The intestinal barrier function requires the complexity of epithelial, which relies on the differentiation of intestinal stem cells (Yu et al., 2020). ACE2 may dictate the stemness of intestinal stem cells by orchestrating calcium perturbation (Yu et al., 2020). For patients in severe or critical condition, the disordered intestinal flora leads to abnormal intestinal inflammation, which affects the gut-lung axis and aggravates the degree of systemic inflammation during the disease (Schuijt et al., 2016; Zhu et al., 2018). Unstable gut mycobiomes and prolonged dysbiosis persisted in patients with COVID-19 after nasopharyngeal clearance of SARS-CoV-2 (Zuo et al., 2020). A. flavus and Aspergillus niger, were detected in fecal samples from patients with COVID-19, even after clearance of SARS-CoV-2 from nasopharyngeal samples and resolution of respiratory symptoms (Zuo et al., 2020). In patients with SARS-CoV-2 infection accompanied by abdominal pain, diarrhea and other gastrointestinal symptoms, the probiotics such as *Lactobacillus* or *Bifidobacterium* in the intestines are significantly reduced (Xu K. et al., 2020). Nutritional support, and supplementation of probiotics can reduce bacterial translocation and secondary intestinal infections (Dhar and Mohanty, 2020). China’s Guidelines for the diagnosis and treatment of novel coronavirus pneumonia (version sixth) mentioned that herbal medicine with prebiotic effect could be used to maintain the intestinal flora homeostasis and prevent secondary bacterial infections.

### Severe Acute Respiratory Syndrome Coronavirus 2’s Mechanism of Effect on Intestinal Mucosa-Associated Immune System

**FIGURE 2:**
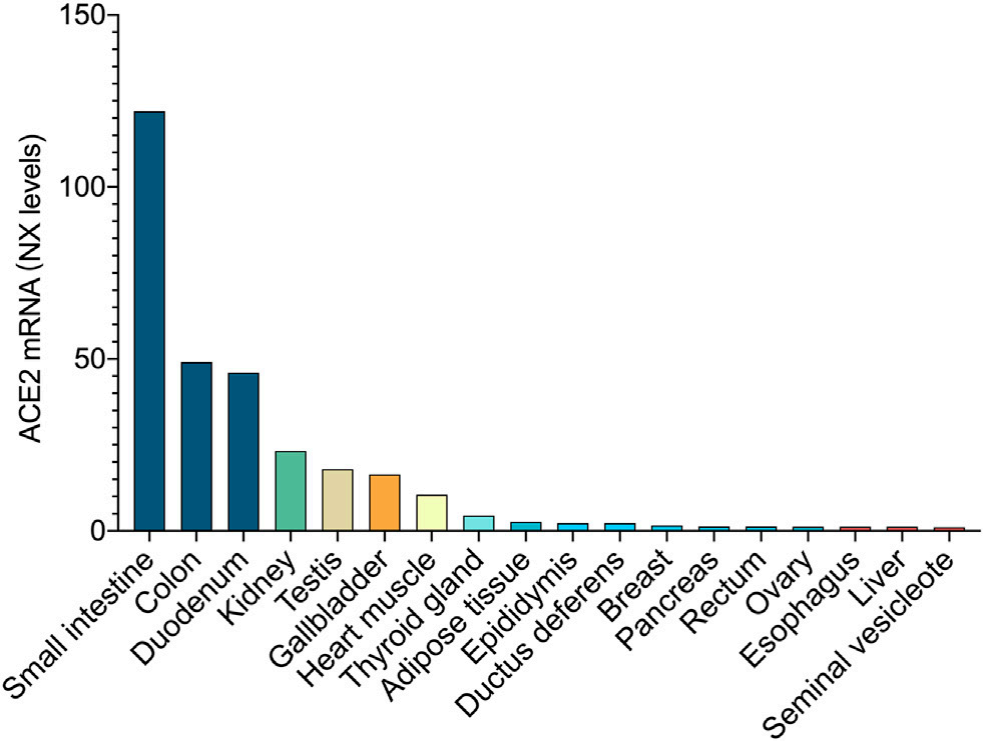
ACE2 expression at the mRNA level. In combined data from Human Protein Atlas, Genotype Tissue Expression, and Functional Annotation of The Mammalian Genome, the top three tissues with the highest expression belong to the intestinal tissue.

The SARS-CoV-2 Spike Glycoprotein can bind to the ACE2 receptor on intestinal epithelial cells’ surface (Walls et al., 2020). ACE2 is expressed in lung cells and intestinal epithelial cells of the esophagus, ileum, and colon. According to the combined data from human protein atlas (http://www.proteinatlas.org), genotype tissue expression and mammalian genome function annotations, the top three tissues with the highest expression levels belong to intestinal tissues (Figure 2). Therefore, SARS-CoV-2 not only infects the respiratory system but may also directly affect the GI system. ACE2 controls the functional expression in the intestines of one of the transport proteins, B0AT1, which acts specifically on neutral amino acids. (Scalise and Indiveri, 2020). The ACE2-B0AT1 exists as a dimer of heterodimers.

Deficiency of ACE2 can also causes a critical impairment of local tryptophan homeostasis which alters the susceptibility to intestinal inflammation (Vuille-dit-Bille et al., 2015). Dietary tryptophan is primarily absorbed via the B0AT1/ACE2 transport pathway on the luminal surface of small intestinal epithelial cells. These differences were reduced when the diet of the ACE2 mutant mice was supplemented with a source of tryptophan (Izcue and Powrie, 2012). Tryptophan has major effects on the host and notably on immunity and metabolism, gut microbiota, intestinal barrier, and transit (Gao et al., 2018; Taleb, 2019). Under normal physiological conditions, the barrier function of the intestinal mucosa is relatively complete, which can effectively prevent the invasion of harmful substances and maintain the stability of the body environment. The intestinal mucosal immune barrier is composed of gut associated lymphoid tissue (GALT) and diffuse immune cells (mainly refers to lamina propria lymphocytes and intraepithelial lymphocytes). GALT can bind to the antigen to produce secretory immunoglobulin A (SIgA). Intestinal mucosal intraepithelial lymphocytes (IEL) and lamina propria lymphocytes (LPL) is the efferent effect and regulatory site of the mucosal immune system (Qiao et al., 1991; Abuzakouk et al., 1998). The stimulated IEL (mainly T lymphocytes) can proliferate rapidly and release a variety of cytokines such as IL-2, IFN-γ, TNF-α. It has the functions of defense against intestinal pathogen invasion, anti-mucosal allergic reaction, suppression of immune response, elimination of damaged epithelial cells and promotion the production of SIgA (Viney and MacDonald, 1990; Olivares-Villagomez and Van Kaer, 2018). LPL (mainly T lymphocytes, B lymphocytes, macrophages, dendritic cells) can secrete a variety of Th2-type cytokines such as TNF, IL-4, IL-6, and IL-10. It contains a large number of plasma cells, which can promote the secretion of Ig A antibodies, neutralize and eliminate antigens. SIgA dimer exists on the surface of the gastrointestinal mucosa and is the main effector molecule of the intestinal mucosal immune response. It can prevent the invasion and adhesion of pathogens, and can bind the corresponding antigen to inhibit bacterial proliferation and neutralize toxins, and can resist proteolytic enzymes. Protect the intestinal mucosa from being digested, and exert local anti-infection and anti-allergic effects (Pal et al., 2013; Zhang J. et al., 2020).

In the absence of ACE2, the expression of the mTOR signaling pathway in the small intestine is reduced, resulting in a decrease in the expression of antimicrobial peptides in the Paneth cells of the small intestine (Hashimoto et al., 2012). The antimicrobial peptides in Paneth cells in the small intestine can change the composition of the intestinal flora and increase intestinal inflammation. Inhibition of the expression of antimicrobial peptides will lead to a sharp increase in the probability of endotoxin and endogenous infection, thereby producing and promoting inflammatory mediators. The cascade reaction caused by the inhibition of the expression of antimicrobial peptides leads to extensive tissue damage, gastrointestinal symptoms, and even multiple organ failure (MOF), leading to a poor prognosis for COVID-19 patients.

As a carboxypeptidase, ACE2 can catalyze Ang Ⅱ to Ang (1-7), which further binds to the cell surface receptor MAS to establish a second axis through ACE2/ANG-(1-7)/MAS, whose end point is the metabolite ANG-(1-7). The downstream ERK, P38, and JNK signaling pathways are regulated by Mas, which plays a protective role in inhibiting the inflammatory response (Passos-Silva et al., 2013). Besides, Ang (1-7) can also directly inhibit or promote the release of nitric oxide from intestinal smooth muscle cells through Mas receptors (Santos et al., 2013), thereby intervening in the activation Ang (1-7) of NF-κB signaling pathways and reducing intestinal inflammatory damage (Souza Santos et al., 2018).

### Restoring Intestinal Flora May Help Prevent and Treat Severe Acute Respiratory Syndrome Coronavirus 2 Infections

Restore the intestinal flora and reduce the intestinal barrier disruption may be of great value in preventing and treating SARS-CoV-2 infection. The intestinal flora can affect the occurrence of respiratory diseases through the production of metabolites (Saint-Georges-Chaumet and Edeas, 2016). A variety of specific microorganisms in the intestine can ferment undigested carbohydrates in the small intestine, produce short-chain fatty acids, and participate in energy metabolism (den Besten et al., 2013; Koeth et al., 2013; Ridaura et al., 2013; Koh et al., 2016). The specific microorganisms can enhance the intestinal epithelial barrier function, immune tolerance, maintain intestinal homeostasis, and reduce the occurrence of infection by down-regulating the expression of inflammatory factors. The intestinal flora can also maintain the ratio of helper T lymphocytes (Th) and reduce airway inflammation (Kao et al., 2020; Kreft et al., 2020). The intestinal flora can promotes the preferential differentiation of anti-inflammatory Treg/Th2 cells while suppressing the relative differentiation of pro-inflammatory Th1/Th17 cells (Li K. et al., 2020). Intestinal flora disorders can cause the dominant Th2 response. In general, the administration of targeted microecological preparations such as non-starch polysaccharides or related herbal medicines may have a specific preventive effect on SARS-CoV-2 infection (Li Y. et al., 2020).

For viral infections, though, overuse of antibiotics is ineffective and increases the patient's exposure to antibiotics in the short term. However, for patients with SARS-CoV-2 infection, especially patients with sepsis, empirical antibacterial treatment can be given within 1 h of the initial evaluation according to the China’s Guidelines for the diagnosis and treatment of novel coronavirus pneumonia (version sixth). However, the colonization resistance of the intestine would be destroyed as soon as antibiotics treatment for pathogenic bacterial infections, caused profound and lasting changes in the intestinal flora. Antibiotics can cause changes in the intestinal flora, which can also lead to an increase in opportunistic pathogens (such as *Clostridium difficile*) and more likely to cause infections. The intestinal flora contains probiotics, which can prevent the colonization of pathogens. Antibiotic treatment destroys the structure of the intestinal flora and reduces resistance to pathogenic bacteria (Knight and Girling, 2003; Li L. et al., 2021). The metabolites of the intestinal flora can also affect the permeability of the host intestinal mucosa barrier (Arpaia et al., 2013). The intestinal mucosal barrier not only participates in the intake of food nutrition and flora metabolites but also has a critical barrier function to prevent microbial invasion and inhibit the inflammatory response to the intestinal contents (Turner, 2009). The intestinal mucosal barrier includes continuous monolayer intestinal epithelial cells and the mucus they secrete. As the recognition site of intestinal microbes, intestinal epithelial cells often interact with microbes and their metabolites to promote the development of the intestinal immune system which participate in immune response and maintain the homeostasis of the intestinal flora (Cario and Podolsky, 2000; Eckburg et al., 2005). The intestinal mucosal immune system is composed of the intestinal epithelium and various secretions on its surface, scattered immune cells, intestinal microbiota, and intestinal-related lymphoid tissues (Hooper et al., 2012). In sepsis, the dysbiosis of intestinal flora will lead to changes in the physiological and anatomical structure of the intestinal mucosal barrier, the proliferation of intestinal cells is significantly reduced, and the apoptosis of intestinal villi and crypt cells is increased dramatically (Weinstein et al., 1975). Intestinal flora and its metabolites play a significant role in maintaining the health of the body.

In patients with SARS-CoV-2 infection, early administration of microecological preparations and prebiotics can restore the balance of intestinal flora and strengthen the intestinal barrier, which is a crucial measure to avoid the aggravation of COVID-19 (Hu et al., 2021). In addition to damages the lungs, SARS-CoV-2 infection cause damage to multiple organs such as the gastrointestinal tract. The change of the intestinal flora is one of its essential pathophysiological mechanisms (Olaimat et al., 2020). The use of prebiotics and herbal medicines with prebiotic effects regulate the intestinal flora, maintain the microecological balance of the gastrointestinal tract, improve the body's immunity, and reduce virus damage to the lungs (Heidari et al., 2021). Prebiotics and herbs with prebiotic effects may help control the progression of severe COVID-19 patients and speed up the recovery process of patients infected with SARS-CoV-2.

## Herbal Medicine, Gut Microbiota and Coronavirus Disease 19

### Herbal Medicines as Angiotensin-Converting Enzyme 2-Blockers in Intestinal Tract

**FIGURE 3:**
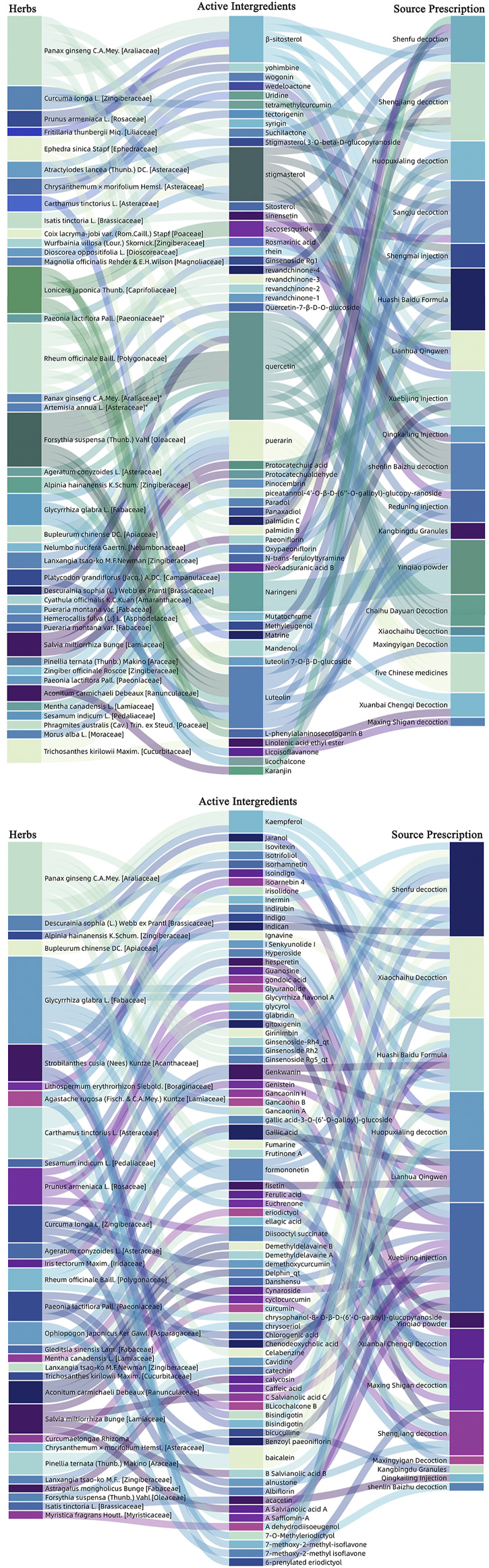
Herbs with potential ACE2 receptor blockers, as well as related active ingredients and derivative prescriptions. We analyzed the most commonly used Chinese medicine prescriptions in the “Diagnosis and Treatment Protocol for Novel Coronavirus Pneumonia.” Herbal medicines with potential ACE2 receptor blockers and related active ingredients have been sorted out.

Angiotensin-converting enzyme 2 (ACE2) efficiently binds the S1 domain of the SARS-CoV S protein. ACE2 is a functional receptor for SARS-CoV. SARS-CoV-2 uses the SARS-CoV receptor ACE2 for entry and the serine protease TMPRSS2 for S protein priming. Several retrospective studies conducted on COVID-19 patients infected with SARS-CoV-2 pointed out that the combination of herbal medicine and Western medicine can significantly improve the clinical symptoms of COVID-19 patients and shorten the treatment time for patients in severe or critical condition (He J. et al., 2020; Lee et al., 2021; Luo et al., 2021). As show in Figure 2, ACE2 is highly expressed in GI tract. Herbs are taken orally and absorbed through the intestine. It is important to understand which herbs and their active ingredients have potential inhibitory effects on ACE2. Here, we list the commonly used herbal prescriptions (Perez-Roses et al., 2015; Saranya et al., 2017; Chuan et al., 2020; Feng et al., 2020; Huang L. et al., 2020; Kim and Kim, 2020; Kim M. et al., 2020; Lin et al., 2020; Liu and Zhang, 2020; Ma J. et al., 2020; Ma Q. et al., 2020; Niu et al., 2020; Qu et al., 2020; Sen et al., 2020; Song et al., 2020; Sun K. et al., 2020; Tao et al., 2020; Wang S. et al., 2020; Wang et al., 2020b; Ya et al., 2020; Yao et al., 2020; Zhang et al., 2020b; Hong et al., 2021; Jia et al., 2021; Li Y. et al., 2021; Lin et al., 2021) in the “China Novel Coronavirus Pneumonia Diagnosis and Treatment Program.” Among them, some herbs with potential ACE2-blockers and their related active ingredients are sorted out in Figure 3. Research results show that the herbs as mentioned above may maintain the balance of RAS-related pathways (Saranya et al., 2017), inhibit the activation of the complement system (Perez-Roses et al., 2015), blunt inflammation (Kim M. et al., 2020; Kim and Kim, 2020), participate in immune regulation (Qu et al., 2020), inhibit the activity of SARS-CoV-2 3C-like protease (Chuan et al., 2020; Feng et al., 2020; Huang L. et al., 2020; Lin et al., 2020; Sen et al., 2020; Tao et al., 2020; Ya et al., 2020), and inhibit the binding process of ACE2 (Hoffmann et al., 2020; Ma Q. et al., 2020; Niu et al., 2020; Song et al., 2020; Wang et al., 2020b).

### Herbal Medicines Restore the Intestinal Flora

As mentioned above, restoring intestinal flora may help prevent and treat SARS-CoV-2 infections. In clinical practice, herbal medicines are usually extracted by water or ethanol and are mainly taken orally. An intestine is an important place for oral drugs to be metabolized in the body (Feng et al., 2019). After the oral medication, they interact with a large number of microorganisms in the intestine (Hamasaki et al., 2000; Feng et al., 2019). Some types of herbal medicines can regulate the intestinal flora’s composition and metabolism, thereby improving the body's dysfunction and pathological conditions. The intestinal flora can participate in the metabolic transformation of herbs, and at the same time, can transform herbal compounds to improve bioavailability (Zuo et al., 2002).

There are five main categories of herbal ingredients that can affect the intestinal flora, including glycoside, flavonoids, alkaloids, phenylpropanoids, and organic acids. The intestinal flora secretes glycoside hydrolases, lyases and esterases to cut sugar chains to obtain energy. An important type of product produced by polyglycolysis is short-chain fatty acids, including acetic acid, propionic acid and butyric acid. Short-chain fatty acids have a wide range of physiological function (Yang et al., 1996; Abdel-Hafez et al., 1998; Bae et al., 2004; Guo et al., 2012; Feng et al., 2015; Xia et al., 2016; Liu et al., 2019; Shao et al., 2019; Wang et al., 2021). Most flavonoids (except flavanols) naturally combine with sugars to form β-glycosides, and only a small part of flavonoids is absorbed by the small intestine. Most of the glycosylated flavonoids will reach the colon and will be broken down into phenolic acid or other metabolites that can be absorbed by the body under the action of the colonic microflora. Flavonoids existing in the colon have a certain regulatory effect on the intestinal flora, and flavonoids catabolized by microorganisms can change their bioavailability and activity (Lee et al., 2004; Xiuwei et al., 2005; Taiming et al., 2006; Knaup et al., 2007; Shi et al., 2009; Trinh et al., 2010; Zhou et al., 2014; Zhou et al., 2015; Xin et al., 2019; Jin et al., 2020b; Wang et al., 2020a). Alkaloids are a type of nitrogen-containing organic compounds derived from the biological world. They have significant physiological activities and are one of the important components of Chinese medicine. Some alkaloids have a certain degree of hydrophilicity and are also soluble in organic solvents. The structural characteristics of these alkaloids are often small molecules, ether bonds, coordination bonds, etc., which are prone to hydrolysis and dehydration reactions under the action of the intestinal flora (Ying et al., 2002; Weiming, 2005; Huaixia et al., 2006; Yufeng et al., 2008). With a lactone structure, phenylpropanoids is easy to undergo biotransformation such as lactone hydrolysis or demethylation under the action of the intestinal flora (Jan et al., 2009; Zhao et al., 2009). A small amount of organic acids is absorbed in the stomach and small intestine as a prototype, and then hydrolyzed under the action of the esterase of the intestinal mucosa and the intestinal flora for further metabolism. Intestinal bacteria can metabolize polyphenols or carbohydrates in the diet to produce organic acids (Weikao et al., 2008; Kim et al., 2013). The production of organic acids is related to *Clostridium, Escherichia coli*, and *Lactobacillus*. Lactic acid can regulate intestinal peristalsis and inhibit the reproduction of harmful bacteria.

Here, we sort out some representative herbs that interact with the intestinal flora according to the types of active ingredients, as well as the possible mechanisms of the interaction of these herbs and effective ingredients with the intestinal flora. The interaction between the effective ingredients of traditional Chinese medicine and the intestinal flora are summarized in Table 1.

**TABLE 1:** The interaction between the effective ingredients of traditional Chinese medicine and the intestinal flora

Category	Representative herbs	Mechanism of herbal absorption and its relationship with the intestine	Main metabolite
Glycoside	1. Panax ginseng C.A.Mey. [Araliaceae; ginseng radix et rhizoma] (Bae et al., 2004; Xia et al., 2016)	The intestinal flora secretes glycoside hydrolases, lyases and esterases to cut sugar chains to obtain energy. An important type of product produced by polyglycolysis is short-chain fatty acids, including acetic acid, propionic acid and butyric acid. Short-chain fatty acids have a wide range of physiological function.	Notoginsenoside R1, Ginsenoside Rg1, Ginsenoside Rg2, Ginsenoside Re, Ginsenoside Rd, Ginsenoside Rb1, Paeoniflorin metabolin I, Glycyrrhetinic acid, Rhein anthrone, Senna aglycone, polysaccharides, dendrobium polysaccharides.
	*2. Panax notoginseng* (Burkill) *F.H.Chen* [notoginseng radix et rhizoma] (Abdel-Hafez et al., 1998)		
	*3. Glycyrrhiza glabra* L. [Fabaceae; glycyrrhizae radix et rhizoma] (Yang et al., 1996)		
	*4. Rheum officinale* Baill. [Polygonaceae; rhei radix et rhizoma], (Yang et al., 1996)		
	*5. Senna alexandrina* var. alexandrina [Fabaceael; sennae folium] (Yang et al., 1996)		
	*6. Portulaca oleracea* L. [Portulacaceae; portulacae herba] (Feng et al., 2015)		
	*7. Dendrobium nobile* Lindl. [Orchidaceae; dendrobii caulis] (Guo et al., 2012)		
	*8. Chrysanthemum × morifolium* (Ramat.) Hemsl. [Asteraceae; chrysanthemi flos] (Wang et al., 2021)		
Flavonoids	*1. Scutellaria baicalensis* Georgi [Lamiaceae; scutellariae radix] (Taiming et al., 2006; Trinh et al., 2010)	Most flavonoids (except flavanols) naturally combine with sugars to form β-glycosides, and only a small part of flavonoids is absorbed by the small intestine. Most of the glycosylated flavonoids will reach the colon and will be broken down into phenolic acid or other metabolites that can be absorbed by the body under the action of the colonic microflora. Flavonoids existing in the colon have a certain regulatory effect on the intestinal flora, and flavonoids catabolized by microorganisms can change their bioavailability and activity.	Baicalein, Oroxylin A, 3,4-Dihydroxybenzoic acid, Gallol and Phenylacetic acid, Kaempferol, Kaempferol glycosides, Parahydroxybenzoic acid, Hesperetin, Equol, Quercetin, gallate, flavanol, pulvin-3-acetate, Epimedium koreanum Nakai-Prenylated Flavonoids, Mulberry leaf flavonoids
	*2. Siraitia grosvenorii* (Swingle) C.Jeffrey ex A.M.Lu & Zhi Y.Zhang [Cucurbitaceae; siraitiae fructus] (Xiuwei et al., 2005)		
	*3. Forsythia suspensa* (Thunb.) Vahl [Oleaceae; forsythiae fructus] (Lee et al., 2004)		
	*4. Glycine max* (L.) Merr. [Fabaceae; sojae semen praeparatum] (Knaup et al., 2007)		
	*5. Epimedium koreanum* Nakai [Berberidaceae; epimedii folium] (Zhou et al., 2014)		
	*6. Morus alba* L. [Moraceae; mori cortex] (Wang et al., 2020a)		
	*7. Hippophae rhamnoides* L. [Elaeagnaceae; hippophae fructus (Xin et al., 2019)		
	*8. Epimedium brevicornu* Maxim. [Berberidaceae; epimedii folium] (Zhou et al., 2015)		
	*9. Sophora flavescens* Aiton [Fabaceae; sophorae flavescentis radix] (Jin et al., 2020b)		
	*10. Coptis chinensis* Franch. [Ranunculaceae; coptidis rhizoma] (Shi et al., 2009)		
Alkaloids	*1. Aconitum carmichaeli* Debeaux [Ranunculaceae; aconiti lateralis radix praeparata] (Ying et al., 2002; Yufeng et al., 2008)	Alkaloids are a type of nitrogen-containing organic compounds derived from the biological world. They have significant physiological activities and are one of the important components of Chinese medicine. Some alkaloids have a certain degree of hydrophilicity and are also soluble in organic solvents. The structural characteristics of these alkaloids are often small molecules, ether bonds, coordination bonds, etc., which are prone to hydrolysis and dehydration reactions under the action of the intestinal flora.	16-O-desmethylaconitine, aconitine, matrine, sinomenine, 3-methoxymorphine, 3 -Methoxy-6hydroxy-17-methylmorphinane, scopolamine, dihydroberberine, berberine, normethyleneberberine, jatrorrhizine, Scopolamine
	*2. Sinomenium acutum* (Thunb.) Rehder & E.H.Wilson [Menispermaceae; sinomenii caulis] (Weiming, 2005)		
	*3. Hyoscyamus niger* L. [Solanaceae; hyoscyami semen] (Huaixia et al., 2006)		
	*4. Coptis chinensis* Franch. [Ranunculaceae; coptidis rhizoma] (Huaixia et al., 2006)		
Phenylpro-panoids	*1. Linum usitatissimum* L. [Linaceae; lini semen] (Jan et al., 2009)	With a lactone structure, it is easy to undergo biotransformation such as lactone hydrolysis or demethylation under the action of the intestinal flora.	Enterodiol, Intestinal Fat, ST-6, arctigenin
	*2. Schisandra chinensis* (Turcz.) Baill. [Schisandraceae; schisandrae fructus] (Jan et al., 2009)		
	*3. Arctium lappa* L. [Asteraceae; arctii fructus] (Zhao et al., 2009)		
Organic acids	*1. Lonicera japonica* Thunb. [Caprifoliaceae; lonicerae japonicae caulis] (Weikao et al., 2008)	A small amount is absorbed in the stomach and small intestine as a prototype, and then hydrolyzed under the action of the esterase of the intestinal mucosa and the intestinal flora for further metabolism. Intestinal bacteria can metabolize polyphenols or carbohydrates in the diet to produce organic acids. The production of organic acids is related to *Clostridium, Escherichia coli*, and *Lactobacillus*. Lactic acid can regulate intestinal peristalsis and inhibit the reproduction of harmful bacteria.	Caffeic acid, quinic acid, ferulic acid, 3-hydroxyphenylpropionic acid, benzoic acid, phenylpropionic acid, hippuric acid derivatives, p-coumarinic acid, chlorogenic acid
	*2. Houttuynia cordata* Thunb. [Saururaceae; houttuyniae herba] (Kim et al., 2013)		
	*3. Eucommia ulmoides* Oliv. [Eucommiaceae; eucommiae cortex] (Kim et al., 2013)		

### Herbal Medicine That Regulates the Intestinal Mucosal Barrier

Stable intestinal tight junction function is an important prerequisite for the stability of the intestinal mucosal barrier. It is closely related to tight junction protein including Occludin, ZO-1, and Claudin, and plays a key role in maintaining intestinal permeability. Tight junction protein is a critical protein that connects the gaps between cells and regulates the intestinal mucosa's permeability. Tight junctions have two main functions: 1) Maintain the polarity of cells, prevent the top and bottom sides of integral membrane proteins from spreading from the side; 2) Prevent ions and molecules from passing through Intercellular space. Under normal circumstances, the tightly connected structure is complete and the function is normal, but the structure and function will be destroyed under pathological conditions. Herbs relieve the destruction of intestinal epithelial cell tight junctions by proinflammatory cytokines (such as LPS, TNF-α, IFN-γ, IL), up-regulating tight expression junction proteins, and enhance the effect of mechanical barriers (Kim et al., 2009; Shah et al., 2010; Li et al., 2017). There are quite a few herbs that can up-regulate Tight junction protein expression and reduce intestinal mucosal permeability.

The active ingredients in the herbal medicine increase the expression level of tight junction protein, improve the ultrastructure of intestinal epithelial cells, up-regulate the ultrastructure of intestinal epithelial cells ZO-1 and Occludin expression, and then repair the colonic epithelial barrier and reduce Intestinal permeability (Lina et al., 2014). Herbs can protect the intestinal mucosal immune barrier by regulating the levels of related cytokines (Pan et al., 2011; Liu et al., 2016). The mechanism may be to regulate lymphocytes, reduce the level of inflammatory factor TNF-α, and improve the intestinal barrier damage (Dai et al., 2009; Zhang et al., 2012). In China, many herbs have been clinically used to treat COVID-19. We searched for herbs that affected TJ protein from the “China Novel Coronavirus Pneumonia Diagnosis and Treatment Program” and the relevant data were shown in Table 2.

**TABLE 2:** Herbs for the treatment of COVID-19 that can affect the expression of tight junction proteins

HERBS	Active ingredient	TJ proteins	Related mechanism
*Kaempferia galanga* L. [Zingiberaceae; kaempferiae rhizoma]	Kaempferol (Suzuki et al., 2011)	ZO-1, ZO-2, occludin, claudin-1, claudin-3, claudin-4	Enhances intestinal barrier function through the assembly of tight junction proteins
*Thesium chinense* Turcz. [Santalaceae]			
*Styphnolobium japonicum* (L.) Schott [Fabaceae; sophorae flos et flos immaturus]			
*Scutellaria baicalensis Georgi [Lamiaceae;scutellariae radix]*	Baicalin (Zhang J. et al., 2021), Baicalein (Li Q. et al., 2021), Wogonin (Chen et al., 2017), Wogonoside (Huang S. et al., 2020)	ZO-1, occludin, claudin-1, JAM-1	Alleviate the down-regulation of tight junction proteins
*Podophyllum versipelle Hance [Berberidaceae]*	Quercetin (Suzuki and Hara, 2009)	ZO-2, occludin, claudin-1, claudin-4	Improving assembly of ZO-2, occludin and claudin-1 enhances intestinal barrier function
*Hypericum ascyron L. [Hypericaceae]*			
*Apocynum venetum L. [Apocynaceae;apocyni veneti folium*]			
*Curcuma longa* L. [Zingiberaceae; curcumae longae rhizoma]	Eucalyptol (Kim D. Y. et al., 2020)	ZO-1, occludin-1	Reversed the induction of tight junction-associated proteins of ZO-1, occludin-1 in glucose-exposed RPE cells
*Ocimum basilicum* L. [Lamiaceae; basilici herba]			
*Amorpha fruticosa* L. [Fabaceae]			
*Paeonia lactiflora Pall. [Paeoniaceae;paeoniae alba radix]*	Paeoniflorin (Wu X.-X. et al., 2019; Cao et al., 2021)	claudin-4, occluding and ZO-1	Protect intestinal barrier by up-regulating the expression of these tight junction proteins
*Paeonia obovate Maxim. [Paeoniaceae], Paeonia anomala subsp. Veitchii (Lynch) D.Y.Hong & K.Y.Pan [Paeoniaceae; paeoniae rubra radix]*			
*Camellia sinensis (L.) Kuntze [Theaceae;camelliae non fermentatum folium]*	Catechin (Wu Q. et al., 2021)	ZO-1	Repair the loose tight junction ZO-1
*Senegalia catechu (L.f.) P.J.H.Hurter & Mabb. [Fabaceae;catechu]*			
*Senegalia catechu (L.f.) P.J.H.Hurter & Mabb. [Fabaceae;catechu]*	Magnolo (Xia et al., 2019)	occludin,	Modulated the expression of occludin
*Curcuma longa L. [Zingiberaceae;curcumae longae rhizoma]*	Curcumin (Wu S. et al., 2021)	ZO-1, occludin, claudin-5	Upregulated the protein expression of ZO-1, occluding and claudin-5
*Alisma plantago-aquatica subsp. Orientale (Sam.) Sam. [Alismataceae;alismatis rhizoma]*	Alisol A 24 (Lu et al., 2021)	ZO-1, claudin-1, occludin-1	Enhanced cell viability and increased ZO-1, claudin-5, and occludin expression
	, Alisol B 23 (Zhu et al., 2021)		
*Senna alexandrina Mill. [Fabaceae;folia sennae]*	Aloe Emodin (Zhang et al., 2020c)	ZO-1 and ZO-2	Restore the expression of the tight junction proteins of ZO-1and ZO-2
*Ocimum basilicum L. [Lamiaceae;bacilici folium]*	Thymol (Omonijo et al., 2019)	ZO-1	Upregulate the expression of crucial proteins of tight junctions to maintain barrier functions
*Ocimum gratissimum L. [Lamiaceae;oleum ocimi gratissimi]*			
*Phellodendron amurense Rupr. [Rutaceae; phellodendri cortex]*	Obacunone (Luo et al., 2020)	TJP1, occludin	Promoted the expression of tight junction proteins (TJP1 and occludin)
*Pueraria montana* var. lobata (Willd.) Maesen & S.M.Almeida ex Sanjappa & Predeep [Fabaceae; puerariae flos]	Puerarin (Li Q. et al., 2021)	ZO-1, occludin	Increase the level of ZO-1 and occludin
*Coptis chinensis* Franch. [Ranunculaceae; coptidis rhizoma]	Berberine (Li Q. et al., 2021)	ZO-1, occludin	Increase the level of ZO-1 and occludin
*Glycyrrhiza glabra* L. [Fabaceae; glycyrrhizae radix et rhizoma]	Glycyrrhiic Acid (Li Q. et al., 2021)	ZO-1, occludin	Increase the level of ZO-1 and occludin
*Panax ginseng* C.A.Mey. [Araliaceae; ginseng radix et rhizoma]	Ginsenoside Rb1, Rg1 (Xu et al., 2019)	occludin	Maintaining the proper assembly of the TJ multiprotein comple
*Panax quinquefolius* L. [Araliaceae; panacis quinquefolii radix]			
*Reseda odorata* L. [Resedaceae]*, Digitalis purpurea* L. [Plantaginaceae; folia digitalis]	Luteolin (Li B.-L. et al., 2021)	ZO-2, claudin-3, claudin-4	The expression of occludin, claudin and ZO1 was increased by luteolin
*Citrus × aurantium* L. [Rutaceae; aurantii fructus immaturus]*, Citrus medica* L. [Rutaceae; citri sarcodactylis fructus]	Naringenin (Noda et al., 2012)	ZO-2, occludin, claudin-1,-3,-4	Increases the cytoskeletal association of ZO-2, occludin, and claudin-1, -3, and -4
*Citrus × limon* (L.) Osbeck [Rutaceae; limonis aetheroleum]	Hesperetin (Noda et al., 2012)	occludin, claudin-1	Increases the level of occludin andclaudin-1 and-3
*Morus alba* L. [Moraceae; mori cortex]	Morin (Noda et al., 2012)	claudin-4	Increase the level of claudin-4
*Euchresta japonica* Hook.f. ex Regel [Fabaceae]	genistein (Noda et al., 2012)	claudin-1	Increase the level of claudin-1
*Dendrobium nobile* Lindl. [Orchidaceae; dendrobii caulis]	Erianin (Zhang T. et al., 2019)	occludin, claudin1	The expression of occluding and claudin1 in protein level were incresed
*Pterocarpus indicus* Willd. [Fabaceae]	Pterostilbene (Serreli et al., 2020)	ZO-1, occludin	Up-regulate the expression of ZO-1 and occludin
*Centella asiatica* (L.) Urb. [Apiaceae; centellae herba]	Asiatic Acid (Wróbel et al., 2020)	ZO-1	Up-regulate the level of ZO-1
*Glycyrrhiza glabra* L. [Fabaceae; glycyrrhizae radix et rhizoma]	Diammonium Glycyrrhizinate (Li Y. et al., 2018)	ZO-1, occludin, claudin-1	Promoted the expression of tight junction proteins
*Conioselinum anthriscoides “Chuanxiong”* [Apiaceae; chuanxiong rhizoma]	Ferulic acid (He S. et al., 2020)	occludin and ZO-1	Increase occludin and ZO-1 protein expression and maintain ZO-1 protein distribution
*Lycopodium japonicum* Thunb. [Lycopodiaceae; lycopodii herba]			
*Actaea cimicifuga* L. [Ranunculaceae; cimicifugae rhizoma]			
*Vanilla planifolia* Andrews [Orchidaceae; vanillae fructus]	Vanillin (Liu X. et al., 2021)	occludin and ZO-1	Upregulation the expression of tight junction protein ZO-1 and occludin
*Ginkgo biloba* L. [Ginkgoaceae; ginkgo semen]	Bilobalide (Zhang H. et al., 2021)	ZO-1, Claudin-3, Occludin	Enhanced the expression of ZO-1, Claudin-3, Occludin

### Herbal Medicines Beneficial to the Intestinal Mucosa-Associated Immune System

In addition to protecting the integrity of the intestinal mucosa, herbal medicine also has a regulatory effect on the intestinal mucosa's immune function with a wide range of immunomodulatory effects. In recent years, extensive research on herbs’ intestinal mucosal immunity has helped to reveal its mechanism of action (Yan et al., 2009; Cai et al., 2018). Many herbal medicines, especially those that can restore intestinal flora, can increase the number of M cells in epithelial cells related to Peel's node follicles, promote the proliferation and activation of lymphocytes of Peel's node, and induce local mucosal immune response (Xu and Du, 2020). In addition, herbal medicine can also increase the content of SIgA, IL-2, and IL-4 cytokines in intestinal mucosal proliferating immune cells, improve the body's immunity (Liu et al., 2003; Allam et al., 2015; Chen et al., 2016), and improve the oxidative stress state of intestinal mucosa (Ghaffari et al., 2018; Ghaffari et al., 2019). The presence of a large number of lymphocytes and cytokines near the intestinal mucosal epithelium is one of the important targets of herbal medicine (Tang and Li, 2014). Lymphocytes are an important part of the immune system. Different lymphocytes have different functions. T lymphocytes participate in cellular immunity, among which regulatory T lymphocytes mainly maintain the homeostasis of intestinal mucosal immunity, and Th17 mainly defends against extracellular bacterial infections and mediates chronic inflammation. B lymphocytes participate in humoral immunity and can differentiate into plasma cells under the stimulation of antigens. NK lymphocytes can directly kill certain target cells. There are a large number of cytokines near the intestinal mucosal epithelium, including lymphokines, interleukin (IL), tumor necrosis factor (TNF), interferon (IFN) and so on (Pitman and Blumberg, 2000). Herbal medicine has a good regulatory effect on intestinal immunity, and can regulate pro-inflammatory cytokines (IL-2, IFN-γ, TNF-α) and anti-inflammatory cytokines (IL-4, IL-5, IL-6, IL-10) Expression (Wu et al., 2003; Yasui and Irahara, 2007).

The secretion balance of pro-inflammatory cytokines and anti-inflammatory cytokines is the guarantee for the body to produce a correct immune response. The immunomodulatory effect of herbal medicine on the body is one of the important mechanisms to prevent and treat intestinal mucosal damage. Herbs can affect the expression of IL-2, IL-4, IFN-γ, and SIg A in the intestinal mucosa, can increase the proliferation of mucosal cells and maintain the integrity of the intestinal mucosa (Zhang et al., 2010; Deng et al., 2018; Zhang M.-X. et al., 2019; Pu et al., 2020; Fang et al., 2021). Herbal medicine, rich in a variety of biologically active ingredients and nutrients, achieves its immunomodulatory effect by activating macrophages, T lymphocytes, B lymphocytes and blood complement proteins. Herbs can reduce the intestinal inflammatory response, reduce intestinal inflammatory factors, and reduce the damage of intestinal epithelial cells by reducing the levels of TNF-α, IL-6 and other inflammatory factors (Zhang, 1984; Ou et al., 2017; Liu et al., 2018; Gao et al., 2019; Zhang H.-Y. et al., 2021; Pang et al., 2021).

### Herbal Medicines Restore Intestinal Flora Which Might Be Effective on Alleviating Specific Complications Caused by Coronavirus Disease 19

#### Acute Respiratory Distress Syndrome and Multiple Organ Dysfunction Syndrome

MODS refers to the occurrence of two or more system or organ dysfunctions or failures at the same time or sequentially after 24 h of acute damage to the body. It is a clinical syndrome in which multiple organ function changes in patients with acute injury cannot maintain a stable internal environment. MODS is dangerous and has a high mortality rate. Among the patients in severe or critical condition after infected by SARS-CoV-2, about two out of three patients will develop severely life-threatening ARDS (Chiumello et al., 2020; Chivato Martin-Falquina et al., 2021), manifested as sudden, stubborn, and rapidly progressing hypoxemia. The mortality rate is much higher. The occurrence of ARDS or MODS may involve the imbalance of intestinal flora (Li et al., 2014; Dickson et al., 2016). The intestines and lungs are closely connected and affect each other. Under stress conditions such as trauma and infection in the body, the intestinal flora is unbalanced, releasing a large amount of active oxygen, and the intestinal barrier function is impaired (Lyte and Bailey, 1997; Souza et al., 2004). The bacteria enter the blood and spread through the blood to other tissues or organs of the host, and finally cause a series of A cascade of inflammatory factors broke out and developed into severe sepsis, leading to ARDS (Anders et al., 2013; Dickson et al., 2016). After the bacterial translocation, the lung is the first organ to be injured, indicating that intestinal infection is the inducement of acute lung injury. Studies have shown that mouse lung microbes transformed from Firmicutes and Proteus to pseudo-nematode community structure (Yajima et al., 2001). In clinical trials, patients with acute respiratory distress syndrome (ARDS) have higher intestinal bacteria levels in bronchoalveolar lavage fluid, which is correlated with the degree of systemic inflammation (Dickson et al., 2016). The primary source of pulmonary flora in sepsis patients may be the lower digestive tract, indicating that the intestinal flora is the bridge between the lungs and the intestines (Dickson et al., 2016). However, the discovery that intestinal flora can cause lung infections may provide new ideas for preventing acute respiratory distress syndrome caused by SARS-CoV-2 infections.

The pathogenesis of MODS is currently unclear. However, the intestine is an important source of bacteria and endotoxins in patients in severe or critical condition, the intestine is one of the important sites for inflammatory cell activation and release of inflammatory mediators (Deng et al., 2001; van der Voort, 2006). For the treatment of MODS, attention should be paid to the prevention and treatment of intestinal dysfunction. Herbal medicine has the following functions in preventing MODS. 1) Herbal medicine can strengthen the effect of enteral nutrition, enhance the body's immune protein synthesis, and more effectively improve the patient's serum protein, muscle, fat and other nutritional indicators (Yongbing et al., 2008; Hu et al., 2011; Pang et al., 2012; Zhang Q. et al., 2020). Early enteral nutrition, especially nutrients with immunomodulatory components, can prevent intestinal mucosal atrophy and reduce the occurrence of bacterial translocation and sepsis. 2) Herbs can regulate the intestinal flora, which can prevent the overgrowth and reproduction of Gram-negative bacteria (Wu Y.-R. et al., 2019; Liu Y.-T. et al., 2021). 3) Herbal medicine has an antioxidant effect and can effectively reduce the damage of oxygen free radicals to cell membranes (Wu Y.-R. et al., 2019; Liu Y.-T. et al., 2021). Herbs can also protect intestinal endothelial cells from oxygen free radical damage, prevent intestinal toxins from entering the blood circulation (Xiyu et al., 2006; Li M. et al., 2018). 4) As mentioned above, herbal medicine can enhance the intestinal barrier function and prevent bacterial translocation.

As the most extensive and crucial functional organ of the GI tract, the intestinal flora is bound to participate in specific complications' occurrence and development. In particular, some abnormal changes in the small intestine found on autopsy suggest the correlation between the SARS-CoV-2 infection and the intestinal flora. Of course, this correlation needs more research to confirm.

#### Sepsis

Sepsis is one of the main causes of death of patients in severe or critical condition, and its pathophysiological mechanism is more complicated. SARS-CoV-2 invades the body, produces pro-inflammatory and anti-inflammatory reactions, and releases a large number of inflammatory mediators to cause sepsis (Shi et al., 1999; Lin et al., 2000). The intestine, as the largest “reservoir of bacteria” in the body, is considered to be the “priming” organ for sepsis-induced multiple organ dysfunction syndrome (Haseeb and Salwen, 2005). The diversity and stability of the intestinal flora can enhance the host's defense capabilities. When sepsis occurs, the intestinal microenvironment changes, leading to pathological changes such as the destruction of intestinal epithelial cells, inflammatory reactions, and the invasion of pathogenic bacteria, which cause local and remote organ damage (Muller-Werdan and Werdan, 2003). After an autopsy, it was found that the intestinal mucosal barrier of patients who died of COVID-19 was damaged (Varga et al., 2020).

Most of the nutrient supply of intestinal epithelial cells comes from direct absorption from the intestinal lumen. The intestinal flora mainly depends on the intestinal nutrients to survive and participate in the metabolism of nutrients (Guarner and Malagelada, 2003). Some polysaccharides and other ingredients in herbal medicine can be metabolized by bacteria into short-chain fatty acids (SFCA)(Rechkemmer et al., 1988; Huo et al., 2020), including butyric acid, acetic acid, propionic acid, etc. Among them, butyric acid is helpful for the repair of intestinal mucosa and the prognosis of sepsis. SFCA can also be combined with G protein-coupled receptor (GPCR) to play a key role in promoting the stability of the intestinal environment and regulating inflammation. It also affects the function of dendritic cells and regulatory T lymphocytes and the secretion of IgA antibodies. Play an anti-inflammatory effect, thereby maintaining intestinal homeostasis. In conclusion, herbal medicine can maintain the intestinal microecological balance, prevent the migration of flora, and avoid the induction of endotoxemia, which is extremely important for the prevention and treatment of sepsis (Varon, 2009; Varon and Varon, 2015; Fan et al., 2020).

### Herbal Medicines Regulate the Immune Function of Other Respiratory Diseases by Restore Intestinal Flora

The gastrointestinal tract is considered the largest immunological organ in the body having a central role in regulating immune homeostasis (Takiishi et al., 2017). The intestinal flora plays a vital role in the function regulation, immune defense, and material metabolism of the human body (Eckburg et al., 2005; Thaiss et al., 2016). The intestinal mucosa has the function of producing immune tolerance and resisting pathogen invasion. Studies have shown that the intestinal flora can activate the TLR2, TLR3, TLR4, TLR7, and TLR9 signaling pathway (Cario and Podolsky, 2000; Szebeni et al., 2008; Heimesaat et al., 2010) and induce regulatory T cells (Treg). Tregs can negatively regulate the intensity and time of immune responses, and their abnormalities can lead to immune imbalance. The intestinal flora can also regulate helper T cells (Th) to induce the activation of neutrophils and intestinal epithelial cells (Wu et al., 2009; Wu et al., 2010; Geuking et al., 2011). Intestinal flora can also stimulate B lymphocytes to produce inhibitory cytokines, thereby inhibiting the occurrence of inflammation. Therefore, intestinal flora can initiate the body’s active immune response to invading microorganisms while maintaining its immune balance.

Gut-Lung Axis intestinal immunity participates in the regulation of lung immunity and systemic immunity. The “gut-lung axis” refers to the fact that the intestinal flora can affect and regulate the lungs’ immunity and function. Intestinal flora can induce inflammation in mice’s lungs through Toll-Like receptor and promote the infiltration of neutrophils (Sato et al., 2020; Liao et al., 2021). T helper 17 cells’ intestinal induction is a critical step in mucosal protection (Gaboriau-Routhiau et al., 2009), which can recruit neutrophils and promote the secretion of antibacterial factors by bronchial epithelium. Immunization of rat intestines by inactivated *atypical Haemophilus influenzae* can simultaneously increase the number of specific Th17 cells in mesenteric lymph nodes and airways (Essilfie et al., 2011; Olliver et al., 2011). The production of specific antibodies in the respiratory tract coincides with the intestinal response to antigen exposure. Therefore, intestinal flora plays a significant role in the lung defense against microbial invasion (Jung et al., 1995; Hooper and Macpherson, 2010). The immune transmission of the gastrointestinal and respiratory tracts can be achieved through mucosal immunity (Akbari et al., 2001; Aujla et al., 2008). It means that in the treatment of COVID-19, the crucial role of intestinal flora in the regulation of the gut-lung axis should not be ignored.

#### Pneumonia

Infectious lung diseases may cause pathological changes through the following two aspects. One is that immune disorders destroy the intestinal microecological disorders, leading to inflammation. As the intestinal cavity is exposed to many exogenous antigens, the immune system must be strictly controlled to maintain a symbiotic relationship with symbiotic bacteria. The host can distinguish beneficial microorganisms from harmful pathogens and establish a healthy microbial community. The mucosal immune system is responsible for removing pathogens. An inappropriate immune response in this process will destroy the intestines' homeostasis, cause microecological disorders, and lead to metabolic dysfunction and local or systemic inflammation (Williams, 2003; van Wijk and Cheroutre, 2010). After mice are infected with the influenza virus, the intestinal flora becomes unbalanced and adaptive immune suppression, further aggravating lung inflammation (Deng et al., 2020). Also, the administration of antibiotics will cause the disturbance of the intestinal microflora, which will last for a long time. Changes in disease status are mainly caused by changing the susceptibility to infection, the colonization of antibiotic-resistant strains in the intestine, and resistance genes (Sartor, 2004; Buffie and Pamer, 2013). The intestinal microecological imbalance can cause the conditional pathogenic bacteria in the intestine to move up to the oropharynx or lower respiratory tract, causing lower respiratory tract infection (Khalmatova, 2006).

#### Chronic Obstructive Pulmonary Disease

The microecological imbalance of the intestinal flora directly or indirectly promotes the occurrence and development, and severity of COPD. A large increase in Gram-negative bacilli will release endotoxin into the blood, and then return to the right atrium via the inferior vena cava, and perfused into the lungs via the pulmonary artery and capillaries. Endotoxin damage promotes the occurrence and development of COPD (Zhou et al., 2019; Hu et al., 2020). The gut microbiota components, especially Gram-negative bacilli, are also the main species of the lung microbiota of COPD patients, and these bacteria can cause acute exacerbations of COPD (Sun Z. et al., 2020). Herbs have beneficial effects in improving symptoms in stable COPD patients over a 3 month treatment period. The potential underlying mechanism may be attributable to the difference in gut microbiota among patients (Hu et al., 2020) and by inhibiting Th17/Treg’s ratio via restore gut microbiota (Peng et al., 2019).

#### Asthma

Changes in microbial composition accompany bacterial and viral respiratory tract infections. Changes in the intestinal flora may promote the immune response dominated by respiratory allergies, thus playing an essential role in respiratory diseases' pathogenesis (Bjorksten et al., 2001). Also, the increase in asthma risk is related to the rise in the number and abundance of *Bacteroides fragilis* and total anaerobes in the intestine (Shi H.-L. et al., 2020). The intestinal composition flora of asthma patients has undergone significant changes. Herbal medicine can regulate the intestinal flora, thereby improving asthmatic airway inflammation (Fang et al., 2019; Le et al., 2020; Xueren et al., 2020). Herbs can also regulate immune function through intestinal flora and are used to treat acute exacerbations of bronchial asthma (Huiyang et al., 2020). By increasing the number of probiotics, reducing the number of harmful flora and restoring the flora’s diversity, herbal medicine has certain advantages and effects in the treatment of intestinal microflora disorders and allergic asthma.

## Conclusion

**FIGURE 4:**
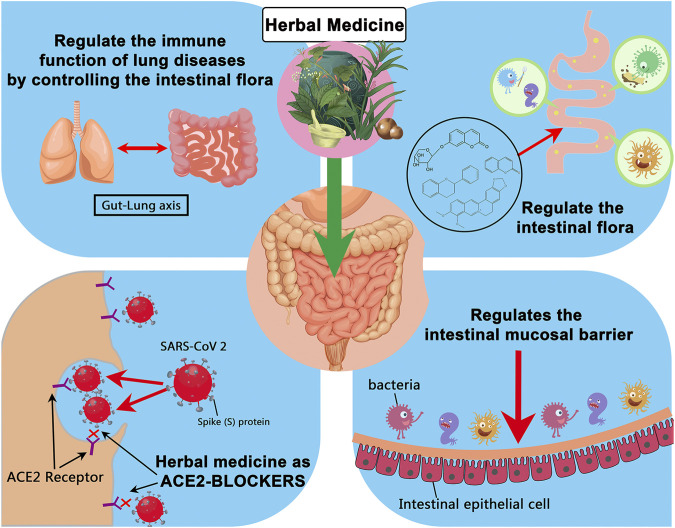
Summary of possible ways in which herbal medicines can affect the prognosis of COVID-19 by regulating the intestinal flora.

In many parts of the world, herbal medicine can be used to regulate and maintain the intestinal flora balance, thereby reducing the incidence of secondary bacterial infections. Due to hypoxia, inflammatory factors, and the use of antibacterial drugs, patients in severe or critical condition will have severe disturbances in the intestinal microenvironment, and critically ill patients are more likely to die from secondary bacterial infections. The “Diagnosis and Treatment Protocol for Novel Coronavirus Pneumonia” (fourth trial edition, trial fifth revised edition, sixth trial edition) [40–42] also mentioned the use of intestinal microecological regulators to maintain the intestinal flora Balance and prevent secondary bacterial infections. Many patients with mild COVID-19 chose to self-isolate at home to enhance their immunity and achieve self-healing. The immune function of patients is essential in defeating and clearing the virus. The gut microbiota in particular plays important roles in host metabolism, immunity and anti-inflammation. Herbs can restore the structure of the intestinal flora, which may further modulate the immune function after SARS-CoV-2 infection. Regulation of intestinal flora by herbal medicine may be helpful for the treatment and recovery of the disease (Figure 4). Understanding the role of herbs that regulate intestinal flora in fighting respiratory virus infections and maintaining intestinal flora balance can provide new ideas for preventing and treating COVID-19.
